# Radiation exposure in the remote period after the Chernobyl accident caused oxidative stress and genetic effects in Scots pine populations

**DOI:** 10.1038/srep43009

**Published:** 2017-02-22

**Authors:** Polina Yu. Volkova, Stanislav A. Geras’kin, Elizaveta A. Kazakova

**Affiliations:** 1 Russian Institute of Radiology and Agroecology, Obninsk, 249032, Russia

## Abstract

Even 30 years after the Chernobyl accident, biological effects of irradiation are observed in the chronically exposed Scots pine populations. Chronic radiation exposure at dose rates above 50 mGy∙yr^−1^ caused oxidative stress and led to the increase of antioxidants concentrations in these populations. Genetic variability was examined for 6 enzymes and 14 enzymatic loci of 6 Scots pine populations. Dose rates over 10 mGy∙yr^−1^ caused the increased frequency of mutations and changes in genetic structure of Scots pine populations. However, the same dose rates had no effect on enzymatic activities. The results indicate that even relatively low dose rates of radiation can be considered as an ecological factor which should be taken into account for ecological management and radiation protection of biota species.

As sessile organisms, plants often exist in unfavorable or even stressful conditions, including abiotic and biotic stresses. Such conditions may disturb metabolism, growth and development of plants^1^,^2^, while studies of plant responses to stress provide important information about the underlying mechanisms of adaptation. The utilization of a stress factor that is easy to measure and which mechanism of action is well-known greatly facilitates the analysis of the adaptation process in natural populations. The ionizing radiation meets both requirements, at least in terms of knowledge of its effects on molecular and cellular levels of biological organization, while an accurate estimation of absorbed dose/dose rate is also possible using specially developed for the experimental object dosimetric models.

There are few sites where the influence of ionizing radiation on plant populations in natural conditions can be studied. The Chernobyl accident is known to be the most severe radiation disaster in the human’s history. The explosion on 26^th^ April 1986 contaminated^3^ more than 200 000 km^2^ with the total released radioactivity of 5 300 PBq^4^. Even now, 30 years after the accident, vast territories remain polluted with radionuclides. Ionizing radiation is a strong mutagenic factor and could possibly have two types of effects: (*i*) direct effects, which include gene mutations and induction of breaks in DNA by energy transfer; (*ii*) and indirect effects, which are caused by reactive oxygen species produced by the radiolysis of water molecules. Radiation-induced mutations can alter the DNA structure hence leading to allelic DNA variations. In this context, electrophoresis of isozymes is extremely useful for quantification of the genetic diversity and may be used for assessment of differentiation between populations growing under different ecological conditions. Mutations in isozyme loci are codominant, thus they can be already identified in seeds of the exposed trees^5^. Meanwhile, the evaluation of enzyme activities helps to understand if the high mutational rates in isozyme loci influence on the physiological state of plants^6^.

In this study we aimed to understand if relatively low levels of radiation exposure (0.03–66.6 mGy∙yr^−1^) cause changes in the genetic structure and antioxidant system of chronically irradiated pine populations. For this purpose we analyzed Scots pine populations that have been growing for 30 years on the heavily contaminated by the Chernobyl accident territories of Russia and Republic of Belarus.

## Methods and Materials

### Test organism

Scots pine (*Pinus sylvestris* L.) was chosen as a test organism for an assessment of the possible effects of radioactive contamination. It is the dominant tree species in North European and Asian boreal forest, and is widespread on the area contaminated by the Chernobyl accident. The reproductive organs of conifers are especially sensitive to radiation exposure because of their complex organization and long generative cycle^7^,^8^. The presence of a haploid endosperm (megagametophyte) in seeds allows direct determination of a haplotype and recessive mutations^5^. Due to its wide distribution and high radiosensitivity, Scots pine is regarded as one of the basic reference species in the modern concept of the radiation protection of the environment^9^.

### Experimental sites

**Figure 1:**
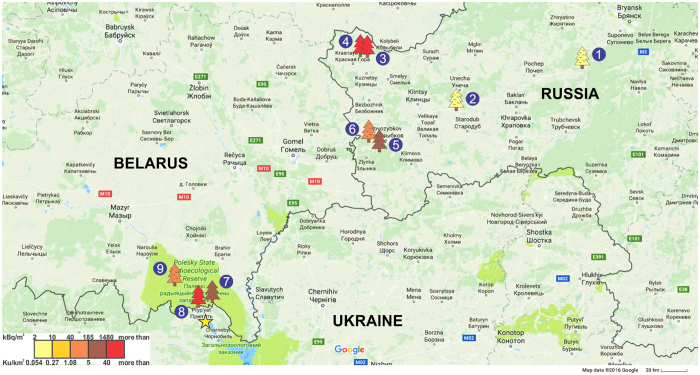
The location of the reference and experimental sites on radioactively contaminated territories. 1 - Ref, 2 - Ref1, 3 - Z1, 4 – Z2, 5 – VIUA, 6 – SB, 7 – Kul, 8 – Mas, 9 – Kozh. The map was created using Google Maps service (the attribution can be seen in the bottom right corner of the Figure) and modified in CorelDraw Graphics Suite X7. Levels of radioactive contamination (in 1998) according to ref. 3.

Our experimental sites are located within the area which was significantly contaminated as a result of the Chernobyl accident. There are two reference and four experimental sites in the Bryansk region of Russia (Fig. 1), and three experimental sites in the territory of Polesskiy Radiation and Ecological Reserve in Republic of Belarus. Two reference sites have been chosen in order to estimate the natural heterogeneity of the experimental populations, and to make it easier to identify if observed changes in the genetic structure and physiological parameters are connected with the level of radiation exposure.

Samples of soil and biological material were taken on each experimental site for estimation of radionuclides and heavy metal concentrations, physical and chemical properties of soils, namely, soil type, pH, humus content, contents of N, P_2_O_5_, K^+^, Ca^2+^, Mg^2+^, cation exchange capacity, hydrolytic acidity. It was found that in terms of physical and chemical properties of soils and heavy metal pollution our sites are quite similar^10^, while the level of radioactive contamination significantly varies from site to site (Table 1). Data received from meteorological stations showed^11^ that moisture and temperature regimes do not differ essentially among experimental sites.

### Dose rate assessment

For the dose rate assessment we used data on radionuclides activities (^137^Cs, ^90^Sr, ^238–241^Pu, ^241^Am) in soil and pine cones, according to the dosimetric model previously developed for calculation of the total (internal+ external) radiation dose absorbed by pine trees crown^10^.

### Sampling

Soil samples were taken at each experimental site at depths of 0–5, 5–10 and 10–15 cm from 3–4 different points under the pine crowns. The different points were merged into one representative sample from each experimental site. Pine cones were collected during December of 2009–2013 from 30 to 50 years old Scots pine trees in Bryansk region (6 experimental sites). At each site, 30–50 cones were taken from 20–29 trees, at a height of 1.5–2.0 m above the ground. Only freely released and well-formed seeds were used for the electrophoretic analyses. Pine needles were collected in November 2015 at the experimental sites in Bryansk region and in Republic of Belarus (9 experimental sites in total). Samples of needles were obtained from 15–18 trees at each experimental site. Five needles were harvested from different sides of each tree, no more than 2.5 m above the ground level. The needles were placed into cryovials and frozen in liquid nitrogen until analysis.

### Electrophoretic analysis of enzyme polymorphism

Six enzymes were chosen for the study of genetic diversity and mutational rates in the experimental populations. Three of them represent the antioxidant system, which is expected to show strong reaction to chronic radiation exposure: superoxide dismutase (EC 1.15.1.1, SOD), glutathione reductase (EC 1.6.4.2, GR), and glutathione peroxidase (EC 1.11.4.2, GPX)^12^. The other three represent different parts of anabolic-catabolic pathways, which are expected to react less evident to chronic radiation exposure, because stability of these systems has high importance for an individual survival: malate dehydrogenase (EC 1.1.1.37, MDH), glucose-6-phosphate dehydrogenase (EC 1.1.1.49, G6PD) and leucine aminopeptidase (EC 3.4.11.1, LAP). The analysis was carried out using 15 endosperms per tree in average. Seeds from different sites were randomized and encoded. Each endosperm was individually homogenized in 100 μl of the extraction buffer (1% triton Х−100 solution and 0.2% solution of β-mercaptoethanol). Supernatants were used for enzyme assays after homogenization and centrifugation (14,500 rpm for 10 min).

Electrophoresis was carried out in vertical chambers «Protean II xi Cell» (USA) and «Hoefer SE 600 Chroma» (USA), using a 7.5% polyacrylamide gel in a Tris-HCl buffer system рН 8.0 with Tris-glycine pH 8.9 as an electrode buffer, for 1.5–2.0 h at 60–80 mA. Gel polymerization took 40–60 min. Isozymes were stained by conventional techniques^13^. Only those bands that could be scored without ambiguity were taken into account. In total, 13,116 loci tests were performed.

Three types of radiation-induced mutations were revealed. Null mutations were identified as an absence of the corresponding allelic variant in the gel. Duplications were manifested by appearance of two enzyme bands in one locus on the gel. Changes in electrophoretic mobility of the isozyme were identified as appearance of the enzyme bands outside the previously identified areas of activity.

### Concentrations of antioxidants

For isolation of the low-molecular weight antioxidants (LMWA) ascorbic acid (AsA), reduced glutathione (GSH), and oxidized glutathione (GSSG), five needles from each experimental tree were grinded with mortar and pestle in liquid nitrogen. The fine powder was then transferred in 1 ml of cold extraction solution (5% H_3_PO_4_, 1 mM EDTA in 0.1% formic acid). The homogenates were centrifuged at 14,500 rpm for 20 min under 4 °C. The supernatants were collected, while the pellets were resuspended with 1 ml of the same extraction solution and centrifuged again under the same conditions. The second supernatant was combined with the first one and filtered through Whatman 0.2 μm nylon membranes with glass microfiber prefilter. For each sample, an aliquot of 500 μl from this mix was stabilized by the addition of 50 μl of 2% butylated hydroxytoluene in ethanol^14^ and stored at −20 °C for the analysis concentration of the oxidative stress reporter malondialdehyde (MDA). The LMWA of the remaining mix was immediately analyzed. All steps were performed at 4 °C with two technical replicates for each sample.

Analyses of LMWA and MDA concentrations were carried out with a high-performance liquid chromatography system Shimadzu LC-30 (Shimadzu, Japan). The analyses were done by injecting of 10 μl aliquots of standard solutions and sample extracts in a reverse-phase columns Shim-pack XR-ODSII (Shimadzu, 2.2 μm, 3.0 × 100 mm, Japan). For LMWA, the autosampler and column temperatures were 6 °C and 30 °C, respectively, while 6 °C and 40 °C were used for MDA. Samples were eluted at a flow rate of 0.5 ml/min.

For LMWA estimation the mobile phase was built using two solvents: solvent A (0.1% formid acid in Milli-Q water) and B (0.1% formic acid in acetonitrile)^15^. For separation of the analytes we used a linear gradient of B from 0 to 10% (0–7 min). Then the column was washed by a linear increase of B concentration from 10 to 90% from 7 to 9 min, being this solvent composition used until 10 min. For the column regeneration, the solvent composition was changed linearly to 0% of B until 12 min, being then was maintained in this state until 17 min. After that a new sample could be injected. Retention times for analytes were 1.7 min (265 nm) for ascorbic acid, 2.1 min (214 nm) for reduced and 3.3 min (214 nm) for oxidized glutathione.

For MDA identification we also used two solvents^14^: solvent A (50 мМ KH_2_PO_4_ in MilliQ water) and B (methanol). For this analysis we used linear gradient from 0–10% of B (0–3 min). Then the column was washed with linear gradient of B from 10 to 90% (3–5 min), being this solvent composition maintained until 6 min. For the column regeneration step the concentration of solvent B was reduced from 90 to 0% (6–8 min), while 100% of A solvent was used until the 11th min. Retention time for MDA standard was 2.4 min (267 nm).

Validation for both methods was provided by obtaining calibration curves, by identification of peaks identities with internal standards, and by estimation of limits of detection. The HPLC system works under control of the software package Lab Solutions (Shimadzu).

### Enzymatic activities analysis

Activities of enzymes (superoxide dismutase, catalase, guaiacol peroxidase, malate dehydrogenase, glucose-6-phosphate dehydrogenase, leucine aminopeptidase) in pine endosperms were assessed^16^ using «NanoDrop-2000» spectrophotometer (USA).

### Statistical analysis

The data are presented as «mean value ± SE». Significance of difference between the means was determined using the t-test. The normal distribution of experimental data was checked prior the statistical analysis. Correlation analysis was done using the Pearson correlation coefficient. We did not apply the multiple comparisons due to using only 2 reference and 7 experimental conditions in our experiment, so its design is different from “case-control” design.

For each population we calculated the allele frequencies and number of indices, which reflect the genetic diversity in experimental populations. An index of the allelic diversity μ and its error s_μ_ were calculated^17^ using (1, 2):


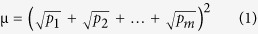


and


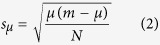


where p_1_, p_2_, … p_m_ are the frequencies of alleles, m is the number of alleles, N – the sample size.

Effective number of alleles (n_e_) was determined as follows (3)^17^:


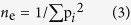


Genetic distances (D) between populations were evaluated as follows (4)^18^:


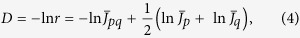


where 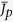 is the averaged over all loci theoretical homozygosity in the first population, 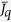 is the averaged over all loci theoretical homozygosity in the second population,.. is the mutual identity of two populations for all loci. The genetic distances matrix was analyzed using cluster analysis by applying the unweighted pair group method (UPGMA) in order to group the populations based on their genetic similarity. Statistical analysis was done using MS Office Excel 2007 software and Statistica 8.0 for Windows.

## Results

### The absorbed doses

The doses annually absorbed by the pine crowns (Table 1) should be regarded as low. However, one should remember that experimental trees had received much higher doses during the first year after the Chernobyl accident, mainly from short-lived radionuclides^19^.

### Isozyme polymorphism

The isozyme analysis revealed an increased mutational rate in chronically irradiated populations (Table 2). The frequency of mutations has shown strong correlation with the level of radiation exposure at the experimental sites (r^2^ = 0.98, p < 0.001).

**Figure 2:**
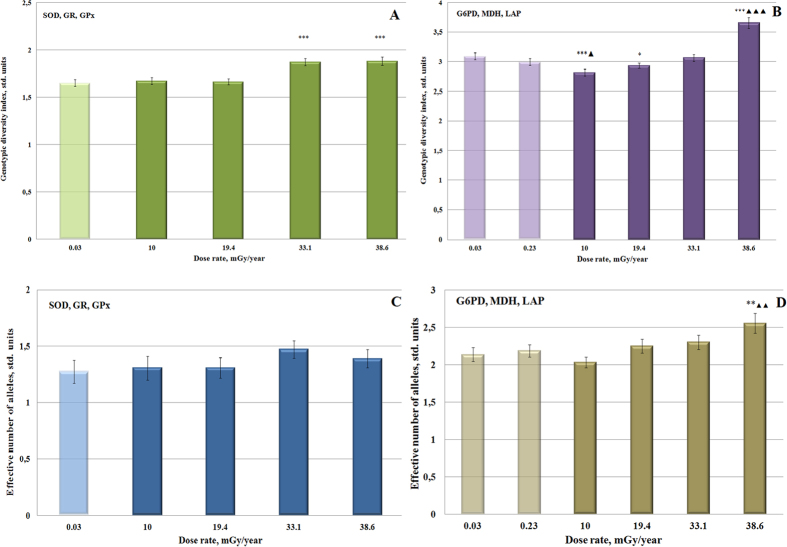
Characteristics of genetic structure of chronically exposed pine populations. Indices of genotypic diversity for antioxidant enzymes (**A**) and for anabolic and catabolic enzymes (**B**). or; Effective numbers of alleles for antioxidant enzymes (**C**) and for anabolic and catabolic enzymes (**D**). T-test revealed a significant difference with: *Ref site, p < 0.05; **Ref site, p < 0.01; ***Ref site, p < 0.001; ^▲^Ref1 site, p < 0.05; ^▲▲^Ref1 site, p < 0.01. ^▲▲▲^Ref1 site, p < 0.001.

All detected loci and allele frequencies of the six evaluated enzymes are presented in Supplementary Table 1. Indices of genotypic diversity (Fig. 2A,B) and effective numbers of alleles (Fig. 2C,D) were calculated for two groups of enzymes. Anabolic and catabolic enzymes are characterized by significantly higher genotype diversity and effective numbers of alleles in comparison with antioxidant enzymes. Considering this, we estimated the mean values of the characteristics of genetic structure of populations separately for each group of enzymes. Interestingly, genotypic diversity of antioxidant enzymes is significantly higher at the most contaminated sites, and shows strong dependence on the absorbed dose (Fig. 2A; r^2^ = 0.94, p < 0.01). Genotypic diversity of anabolic and catabolic enzymes shows much weaker correlation with radiation exposure (Fig. 2B; r^2^ = 0.65, p > 5%). On the other hand, the effective number of alleles for antioxidant enzymes had weak correlation with the dose rate (Fig. 2C; r^2^ = 0.66, p > 5%), while anabolic and catabolic enzymes showed stronger correlation with this value and radiation exposure (Fig. 2D; r^2^ = 0.81, p < 0.01).

**Figure 3:**
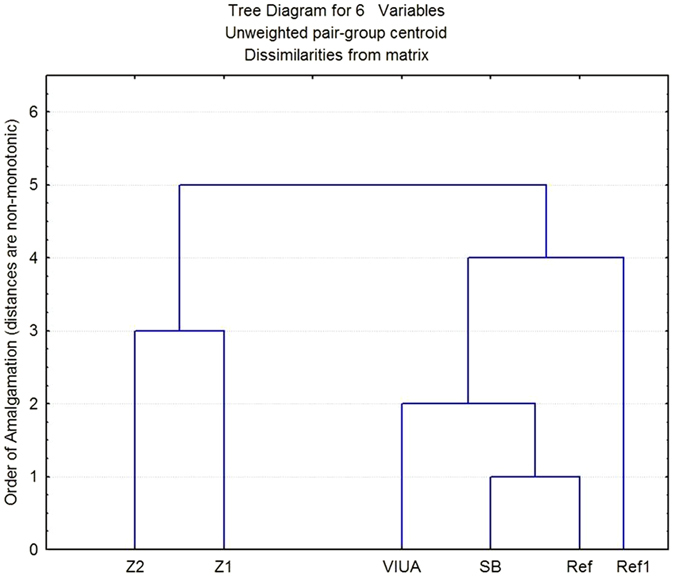
The diagram of genetic distances among 6 experimental populations from radioactively contaminated territories of Russia.

Based on the information about presence and frequencies of alleles, the analysis of genetic distance between experimental populations showed two well-defined groups: the first one included the populations inhabiting heavily contaminated sites (Z1 and Z2), and the second one included much less contaminated sites (Ref, Ref1, VIUA, and SB) (Fig. 3).

### Enzyme activities

Information about biological activity of enzymes is essential for understanding how a gene function and a genetic variation affect phenotypes^6^. In this experiment, significant changes in the activities were found only for POD and G6PD (Table 3). The activities of the remaining four enzymes (SOD, CAT, MDH, and LAP) did not depend on the absorbed dose at all (r^2^ = 0.01–0.45, p < 0.05). While POD activity decreased with the dose rate (r^2^ = 0.79, p < 0.01), the activity of G6PD increased (r^2^ = 0.77, p < 0.01).

### Low-weight antioxidant molecules quantification

Concentrations of low molecular weight antioxidants did not show (Table 3) any significant dependence on the overall level of radiation exposure (r^2^ = 0.20, p > 5% for GSH, r^2^ = 0.20, p > 5% for GSSG, r^2^ = 0.02, p > 5% for AsA). Nevertheless, GSH concentration significantly increased at the most contaminated sites, while GSSG showed an inverse pattern. More interesting results are revealed by the analysis of the GSH/GSSG ratio, which is essentially higher for the most contaminated sites and shows significant correlation (r^2^ = 0.446, p < 0.05) with the level of radiation exposure. Analysis of MDA concentrations showed that experimental populations have undergone the oxidative stress (r^2^ = 0.397, p < 0.05), which is especially evident for the most exposed population Kul (Table 3).

## Discussion

Currently, international organizations are developing a new approach for radiation protection of biota species. There is yet a sufficient lack of field data about the real consequences of chronic radiation exposure for the plant and animal populations^20^. The existing estimates are quite inaccurate and are often based on scientific consensus, while the real experimental data from natural conditions are needed for development of the effective management strategy for radioactively polluted areas. For instance, United Nations Scientific Committee on the Effects of Atomic Radiation considers 100 μGy∙h^−1^ as safe dose rate for plant ecosystems^21^, but even significantly lower dose rates in our study already induce the number of biological effects in populations of Scots pine - the species that can be considered as the main and the most radiosensitive component of a coniferous forest ecosystem (Tables 2 and 3, Fig. 2). Our results suggest that the effective radiation protection approach should be based on the information about effects on populations of reference species and should include different dose thresholds for different types of reference ecosystems.

The studied Scots pine populations have been growing under chronic radiation exposure for almost 30 years. Initially, the levels of radioactive contamination at the experimental sites were high enough^19^ to induce strong biological effects because of the short-lived radioisotopes. Observed biological effects in our study most likely have two reasons: initial high-dose irradiation, and the long-term chronic exposure, which diminishes each passing year. Both of them could probably result in differential gene expression patterns of irradiated individuals. In this context, our experimental sites provide the unique opportunity to study the initial steps of genetic differentiation in natural populations under severe stressful conditions. Changes in genetic structures have been detected in response to strong environmental stress for a number of plant species including forest trees: industrial pollution for *Pinus sylvestri*s^22^ and *Picea abies*^23^; water and nutrient stress for *Pinus edulis*^24^; ozone for *Pinus ponderosa*^25^; temperature for *Betula pendula*^26^. It is known that high level of radiation exposure can also potentially lead to rapid changes in the genetic structure of plant and animal populations^27^, and in our study we consider the increased mutational rate as a potential engine of genetic changes in chronically irradiated pine populations (Table 2). We discovered that some indices of genetic diversity were increasing along the level of radiation exposure (Fig. 2) and frequencies of different alleles significantly varied from site to site (Supplementary Table 1). Genetic differentiation of experimental populations apparently is driven by radiation exposure, especially in the heavy contaminated sites (Fig. 3). Two studied groups of enzymes showed different response to radiation exposure. Antioxidant enzymes play an important role in the ability of population to withstand oxidative stress, induced by chronic irradiation. Possible variations in their allelic structure may be less risky for population as the alterations in the allelic structure of anabolic and catabolic enzymes. Indeed, the genotypic diversity of antioxidant group on the contaminated sites rises faster in comparison with MDH, G6PD and LAP, which even showed a decrease of genotypic diversity at the less contaminated areas in comparison with reference sites. Effective number of alleles, as expected, is higher in the group of anabolic and catabolic enzymes, because loci of these enzymes have very high allelic variability even in the reference groups. However, there were no significant changes in the activities of six evaluated enzymes (Table 3) that suggests that the dose rate in the range of 0.03–38.6 mGy∙yr^−1^ is insufficient to induce an essential biological effect at this level of organization.

The analysis of antioxidants and MDA concentrations showed that even low-dose chronic radiation exposure contributes to the oxidative stress in natural populations (Table 3). MDA, which is a decomposition product of polyunsaturated fatty acids in membranes, is considered to be an indicator of free radical stress. The increase of MDA concentrations indicates a distinct deterioration of membrane integrity and activation of lipid peroxidation in plant cells. The MDA concentrations are increased at radioactively contaminated sites and show slight dependence on the level of radiation exposure (Table 3). In spite of the increased levels of MDA, our study did not reveal any significant changes in the free AsA concentrations in the affected plant populations (Table 3). The ascorbate concentration fluctuates depending on many factors such as light intensity, age, plant tissue, and cell compartment^28^. Probably, more detailed investigation of reduced and oxidized forms of AsA and their ratio in the experimental populations may give more valuable information about the role of this antioxidant in response to chronic low-dose radiation exposure.

Glutathione is an important component of the defense system in plants and animals. Under stress it accumulates in cells, protecting them to oxidative stress by reducing H_2_O_2_. Glutathione is therefore a key factor for cellular redox homeostasis and tolerance against abiotic and biotic stresses, and mainly exists in two forms – reduced (GSH) and oxidized (GSSG) glutathione. The accumulation of glutathione and other antioxidants is commonly observed in plants under stress, as a protective response that may determine whether stress causes only slight and temporary deviations from the normal state, or more severe and permanent damage^29^. The changes of the ratio GSH/GSSG during the degradation of H_2_O_2_ is important in certain redox signaling pathways^30^. Increased glutathione concentrations and GSH/GSSG ratio at the most contaminated sites can be considered as an adaptive reaction. Therefore, the increase of GSH concentration may lead to enhancement of stress tolerance in the chronically irradiated plant populations.

The connection between isozyme polymorphism and an activity of antioxidant system in plants under stress conditions was shown in a few works^31^,^32^. It suggests that some changes observed in the antioxidant system may be due to alterations in the genetic structure of studied populations. In the same time, the main difficulty of our work is to separate the effects of primary high-dose irradiation and of current chronic low-dose exposure. We assume that at least some effects (the increased rate of null-mutations and the high level of oxidative stress at the most contaminated sites) could be connected with the present levels of chronic exposure. Our previous work showed^33^ that the spectrum of chromosomal abnormalities at the most contaminated sites (chromosome lagging, multipolar mitoses) suggested their radiation nature. The maintenance of high MDA concentrations also gives evidence of the heightened concentrations of reactive oxygen species in plant cells, which probably provoke the response of the antioxidant system showed in this research.

Natural ecosystems are often polluted with a mixture of pollutants that can cause a synergetic influence of different stressors on an ecosystem. Different biotic and abiotic factors may interact with pollutants, also causing synergetic effects. A number of works showed synergetic interaction between ionizing radiation and heavy metals: γ-radiation and Cr^34^, γ-radiation and Ni/As^35^, γ-radiation and Cd/Pb^36^. Based on knowledge of possible coaction of ionizing radiation and heavy metals, we carefully chose the experimental sites and made sure that there was no excess of heavy metal concentrations in soil. Another possible input in the observed biological effect could be made by weather conditions and UV-radiation, which is known to cause DNA-damage and interacts synergistically with ionizing radiation^37^. To eliminate this factor the experimental sites were chosen taking into account weather conditions; moreover, the multiple regression analysis of different weather factors (i.e. humidity, the average temperatures, the amount of precipitation etc) did not show any interaction of them with the level of radiation exposure at the experimental sites^11^.

Overall, this study has shown that it is important to estimate the reaction of living things to anthropogenic exposure using different approaches at different levels of biological organization. Strong biological effects at the molecular level of organization turn into moderate effects at the physiological level and into slight effects at the organismal level^10^,^38^. The effect of low-dose chronic irradiation on individuals has remained a matter of discussions, but from the standpoint of our research even quite low doses of radiation exposure influence on a genetic structure of natural population through an increase in the mutational rates. Higher dose rates (starting from 51 mGy∙yr^−1^ in this research) already maintain a higher level of oxidative stress and cause the response of antioxidant system. Recently we have started a new experiment on the first generation of irradiated pine trees (non-exposed in the acute period of disaster, age <25 years), evaluating gene expression profiling. We hope that this data will help to separate biological effects of acute exposure from chronic exposure effects.

## Conclusions

Studies of the mechanisms of adaptation to environmental stress create a scientific basis for developing the strategy of biota protection from man-made impact. The present work is a part of the continuing long-term (2003–2016) study of the biological effects in Scots pine populations, showing that affected Scots pine populations are characterized by an increased level of cytogenetic abnormalities in root meristem of seedlings^10^. However, the higher rate of mutations had no effect on the reproductive ability of these trees, which is largely determined by weather conditions^11^. It follows from this work that relatively low levels of radiation exposure (up to 39 mGy∙yr^−1^) do not significantly change studied physiological parameters in Scots pine populations, but they lead to the occurrence of higher mutational rates and changes in the genetic structure of populations in comparison with the reference sites. At the most contaminated experimental sites (51–66 mGy∙yr^−1^), with the higher contribution of β-radiation to annual dose, we observe difference in the antioxidant status and higher level of oxidative stress compared to the reference sites. Consequently, plant populations remain a great object for study of long-term biological consequences of radiation exposure even 30 years after the Chernobyl accident.

## Additional Information

**How to cite this article:** Volkova, P. Y. *et al*. Radiation exposure in the remote period after the Chernobyl accident caused oxidative stress and genetic effects in Scots pine populations. *Sci. Rep.* **7**, 43009; doi: 10.1038/srep43009 (2017).

**Publisher's note:** Springer Nature remains neutral with regard to jurisdictional claims in published maps and institutional affiliations.

## Supplementary Material

Supplementary Table 1

## Figures and Tables

**Table 1:** The active concentrations (AC) of radionuclides and the annual dose rate (2015) at the experimental sites.

Experimental site	Depth, cm	AC of ^137^Cs in soil	AC of ^137^Cs in cones	AC of ^90^Sr in cones	Annual dose rate, mGy∙yr^−1^
		Bq∙kg^−1^			
Ref^RF^	0–5	13.2 ± 2.6	4.2 ± 2	1.1 ± 2.3	0.02
	5–10	16.2 ± 2.8			
Ref1^RF^	0–5	156 ± 19.8	12.6 ± 3.8	0.8 ± 1.3	0.23
	5–10	127 ± 12.2			
VIUA^RF^	0–5	10800 ± 810	207 ± 26	11.3 ± 2.4	10.0
	5–10	778 ± 65			
	10–15	417 ± 33			
SB^RF^	0–5	13000 ± 1580	302 ± 39	35.9 ± 4.4	19.4
	5–10	12200 ± 1480			
	10–15	653 ± 80			
Z1^RF^	0–5	35600 ± 2992	2170 ± 266	43.2 ± 2.8	33.1
	5–10	4350 ± 405			
	10–15	1120 ± 136			
Z2^RF^	0–5	46200 ± 3243	1420 ± 176	48.7 ± 3.7	38.6
	5–10	4890 ± 340			
	10–15	1460 ± 117			
Kozh^RB^	0–5	3142 ± 33	3200 ± 380	6295 ± 53	18.0
	5–10	865 ± 17			
	10–15	296 ± 9			
Mas^RB^	0–5	66300 ± 820	5470 ± 666	1050 ± 12.6	50.9
	5–10	12900 ± 200			
	10–15	2680 ± 33			
Kul^RB^	0–5	41700 ± 330	14000 ± 1700	4246 ± 40	66.6
	5–10	3823 ± 71			
	10–15	1358 ± 29			

^RF^Russian Federation; ^RB^Republic of Belarus.

**Table 2:** The frequency of mutations in endosperms of chronically irradiated pine trees.

Experimental site	Number of locus-tests	Null mutation frequency	Duplications frequency	Changes in electrophoretic mobility frequency	Total frequency of mutations
Ref	2457	0.002 ± 0.001	0	0.001 ± 0.001	0.003 ± 0.001
Ref1	999	0.003 ± 0.002	0.001 ± 0.001	0.004 ± 0.002	0.008 ± 0.002
VIUA	2459	0.005 ± 0.001	0.001 ± 0.001	0.003 ± 0.001	0.009** ± 0.002
SB	2313	0.011***^▲^ ± 0.002	0.004*** ± 0.001	0.004* ± 0.001	0.020***^▲▲^ ± 0.004
Z1	2589	0.024***^▲▲▲^ ± 0.003	0.007***^▲^ ± 0.002	0.007*** ± 0.002	0.041***^▲▲▲^ ± 0.004
Z2	2228	0.029***^▲▲▲^ ± 0.004	0.011***^▲▲▲^ ± 0.002	0.010***^▲▲^ ± 0.002	0.054***^▲▲▲^ ± 0.050

t-test revealed a significant difference with: *Ref site, p < 0.05; **Ref site, p < 0.01; ***Ref site, p < 0.001%; ^▲^Ref1 site, p < 0.05; ^▲▲^Ref1 site, p < 0.01; ^▲▲▲^Ref1 site, p < 0.001.

**Table 3:** Antioxidant concentrations and enzyme activities in chronically irradiated Scots pine populations.

Experimental site	Dose rate, mGy∙yr^−1^	Concentrations, mM					Enzyme activities, IU					
		GSH	GSSG	AsA	GSH/GSSG	MDA	SOD	CAT	POD	G6PD	MDH	LAP
Ref^RF^	0.02	0.039 ± 0.011	0.018 ± 0.003	0.131 ± 0.025	2.299 ± 0.662	1.286 ± 0.328	0.007 ± 0.006	0.053 ± 0.017	0.409 ± 0.038	0.051 ± 0.025	0.097 ± 0.030	0.017 ± 0.017
Ref1^RF^	0.23	0.032 ± 0.006	0.028 ± 0.004	0.133 ± 0.013	1.357 ± 0.225	1.232 ± 0.229	—	—	—	0.062 ± 0.026	0.112 ± 0.032	0.030 ± 0.030
VIUA^RF^	10.0	0.050 ± 0.015	0.024 ± 0.004	0.141 ± 0.012	1.809 ± 0.455	2.091 ± 0.438	0.012 ± 0.008	0.093 ± 0.022	0.34 ± 0.035	0.061 ± 0.026	0.134 ± 0.035	0.017 ± 0.017
SB^RF^	19.4	0.097*^▲^ ± 0.035	0.025 ± 0.005	0.151 ± 0.014	4.017^▲▲^ ± 0.748	2.143 ± 0.531	0.007 ± 0.007	0.115* ± 0.028	0.39 ± 0.043	0.051 ± 0.025	0.097 ± 0.030	0.021 ± 0.021
Z1^RF^	33.1	0.035 ± 0.010	0.023 ± 0.002	0.125 ± 0.016	1.604 ± 0.682	1.301 ± 0.456	0.006 ± 0.006	0.080 ± 0.025	0.31* ± 0.042	0.083 ± 0.035	0.145 ± 0.036	0.032 ± 0.032
Z2^RF^	38.6	0.050^▲^ ± 0.006	0.030* ± 0.004	0.145 ± 0.016	1.750 ± 0.181	1.154 ± 0.197	0.005 ± 0.005	0.100 ± 0.025	0.15*** ± 0.03	0.084 ± 0.039	0.084 ± 0.028	0.009 ± 0.009
Kozh^RB^	18.0	0.023 ± 0.004	0.022 ± 0.004	0.086^▲▲^ ± 0.008	1.003 ± 0.138	1.044 ± 0.132	n/e	n/e	n/e	n/e	n/e	n/e
Mas^RB^	50.9	0.061^▲^ ± 0.010	0.015^▲^ ± 0.002	0.205 ± 0.061	4.285^▲▲^ ± 0.900	2.000 ± 0.289	n/e	n/e	n/e	n/e	n/e	n/e
Kul^RB^	66.6	0.079*^▲^ ± 0.015	0.015^▲^ ± 0.002	0.107 ± 0.010	6.095*^▲▲^ ± 1.491	3.550***^▲▲▲^ ± 0.321	n/e	n/e	n/e	n/e	n/e	n/e

n/e – not evaluated; ^RF^Russian Federation; ^RB^Republic of Belarus.

t-test revealed a significant difference with: *Ref site, p < 0.05; ***Ref site, p < 0.001; ^▲^Ref1 site, p < 0.05; ^▲▲^Ref1 site, p < 0.01; ^▲▲▲^Ref1 site, p < 0.001.
