# Supplementary material for: Radiation exposure in the remote period after the Chernobyl accident caused oxidative stress and genetic effects in Scots pine populations
Source: Sci Rep. 2017 Feb 22;7:43009. doi: 10.1038/srep43009 (PMC5320440; doi:10.1038/srep43009)
Supplement: Supplementary Table 1 [file srep43009-s1.pdf]

Radiation exposure in the remote period after the Chernobyl accident caused oxidative stress and genetic effects in Scots pine populations

Polina Yu. Volkova[1*], Stanislav A. Geras'kin[1], Elizaveta A. Kazakova[1]

[1]Russian Institute of Radiology and Agroecology, Obninsk, 249032, Russia

*volkova.obninsk@gmail.com

Supplementary Table 1. Loci and allelic frequencies of six investigated enzymes in chronically irradiated plant populations.

Enzyme	Locus	Number of alleles	Allele	Experimental site	Frequency of the allele
SOD	*sod-1*	2	1.00	Ref	1 – 0.063
				VIUA	1 – 0.077
				SB	1 – 0.067
				Z1	0.704*** ± 0.030
				Z2	0.783** ± 0.027
			1.10	Ref	0 + 0.056
				VIUA	0 + 0.066
				SB	0 + 0.059
				Z1	0.296*** ± 0.030
				Z2	0.217*** ± 0.027
	sod-2	1	1.00	Ref	1 – 0.063
				VIUA	1 - 0.077
				SB	1 - 0.067
				Z1	1 – 0.063
				Z2	1 – 0.063
	sod-3	1	1.00	Ref	1 – 0.063
				VIUA	1 - 0.077
				SB	1 - 0.067
				Z1	1 – 0.063
				Z2	1 – 0.063
GPx	*gpx-1*	2	1.00	Ref	1 – 0.063
				VIUA	1 – 0.063
				SB	0.910 ± 0.021
				Z1	0.893 ± 0.021
				Z2	0.906 ± 0.018
			0.80	Ref	0 + 0.056
				VIUA	0 + 0.056
				SB	0.090 ± 0.021
				Z1	0.107 ± 0.021
				Z2	0.094 ± 0.018
	gpx-2	3	1.00	Ref	0.905 ± 0.020
				VIUA	0.905 ± 0.020
				SB	0.880 ± 0.024
				Z1	0.802** ± 0.027
				Z2	0.822* ± 0.024
			0.95	Ref	0 + 0.056
				VIUA	0 + 0.056
				SB	0 + 0.063
				Z1	0.077 ± 0.018

				Z2	0.066 ± 0.016
			1.20	Ref	0.095 ± 0.020
				VIUA	0.095 ± 0.020
				SB	0.120 ± 0.024
				Z1	0.122 ± 0.016
				Z2	0.122 ± 0.020
G6PD	*g6pd-1*	3	1.00	Ref	0.957 ± 0.030
				Ref1	0.944 ± 0.024
				VIUA	0.928 ± 0.020
				SB	0.886 ± 0.038
				Z1	1▲ – 0.167
				Z2	0.735***▲▲▲ ± 0.047
			0.98	Ref	0.043 ± 0.030
				Ref1	0.056 ± 0.024
				VIUA	0.024 ± 0.012
				SB	0.085 ± 0.033
				Z1	0▲ + 0.124
				Z2	0.108 ± 0.033
			1.05	Ref	0 + 0.077
				Ref1	0 + 0.077
				VIUA	0.048 ± 0.017
				SB	0.029 ± 0.020
				Z1	0 + 0.124
				Z2	0.157 ± 0.039
	g6pd-2	3	1.00	Ref	0.848 ± 0.030
				Ref1	0.903 ± 0.028
				VIUA	0.959** ± 0.015
				SB	0.812▲ ± 0.038
			0.95	Ref	0.062 ± 0.020
				Ref1	0.062 ± 0.023
				VIUA	0.012**▲ ± 0.008
				SB	0.148▲ ± 0.035
			1.10	Ref	0.090 ± 0.024
				Ref1	0.035 ± 0.017
				VIUA	0.029 ± 0.013
				SB	0.040 ± 0.019
GR	*gr-1*	2	1.00	Ref	0.848 ± 0.024
				VIUA	0.825 ± 0.025
				SB	0.818 ± 0.028
				Z1	0.815 ± 0.026
				Z2	0.831 ± 0.024
			1.10	Ref	0.152 ± 0.024

				VIUA	0.175 ± 0.025
				SB	0.182 ± 0.028
				Z1	0.185 ± 0.026
				Z2	0.169 ± 0.024
LAP	*lap-1*	11	1.00	Ref	0.122▲▲▲ ± 0.022
				Ref1	0.333*** ± 0.033
				VIUA	0.338*** ± 0,032
				SB	0.187 ± 0.027
				Z1	0.332*** ± 0.032
				Z2	0.266** ± 0.036
			0.95	Ref	0.023▲ ± 0.010
				Ref1	0.076* ± 0.019
				VIUA	0.106** ± 0.021
				SB	0.057 ± 0.016
				Z1	0.166***▲ ± 0.025
				Z2	0.068* ± 0.021
			1.05	Ref	0.201▲▲ ± 0.027
				Ref1	0.081** ± 0.019
				VIUA	0.301*▲▲▲ ± 0.031
				SB	0.278 ± 0.031
				Z1	0.238▲▲▲ ± 0.028
				Z2	0.333*▲▲▲ ± 0.039
			1.10	Ref	0.407▲▲ ± 0.034
				Ref1	0.247** ± 0.031
				VIUA	0.148***▲ ± 0.024
				SB	0.277* ± 0.031
				Z1	0.193*** ± 0.026
				Z2	0.157***▲ ± 0.030
			1.15	Ref	0.121 ± 0.022
				Ref1	0.152 ± 0.025
				VIUA	0.046*▲▲▲ ± 0.014
				SB	0.114 ± 0.022
				Z1	0.040**▲▲▲ ± 0.013
				Z2	0.061*▲ ± 0.020
			1.20	Ref	0.084 ± 0.019
				Ref1	0.086 ± 0.020
				VIUA	0.037*▲ ± 0.013
				SB	0.077 ± 0.018
				Z1	0.018**▲▲ ± 0.009
				Z2	0.054**▲▲ ± 0.019
			1.25	Ref	0.005 ± 0.005
				Ref1	0.005 ± 0.005

				VIUA	0.014 ± 0.008
				SB	0 + 0.056
				Z1	0 + 0.056
				Z2	0.020 ± 0.012
			1.30	Ref	0.014 ± 0.008
				Ref1	0 + 0.056
				VIUA	0.005 ± 0.005
				SB	0.010 ± 0.008
				Z1	0.009 ± 0.006
				Z2	0.020 ± 0.012
			1.35	Ref	0.009 ± 0.006
				Ref1	0.010 ± 0.007
				VIUA	0 + 0.056
				SB	0 + 0.056
				Z1	0 + 0.056
				Z2	0.014 ± 0.010
			1.40	Ref	0 + 0.056
				Ref1	0.010 ± 0.007
				VIUA	0.005 ± 0.005
				SB	0 + 0.056
				Z1	0 + 0.056
				Z2	0.007 ± 0.007
			1.45	Ref	0.014 ± 0.008
				Ref1	0 + 0.056
				VIUA	0 + 0.056
				SB	0 + 0.056
				Z1	0.004 ± 0.004
				Z2	0 + 0.056
MDH	*mdh-1*	8	1.00	Ref	0.276 ± 0.032
				Ref1	0.239 ± 0.030
				VIUA	0.263 ± 0.030
				SB	0.279 ± 0.033
				Z1	0.311 ± 0.032
				Z2	0.331 ± 0.042
			0.90	Ref	0.005▲▲▲ ± 0.005
				Ref1	0.098*** ± 0.021
				VIUA	0.086▲▲▲ ± 0.019
				SB	0.047*▲ ± 0.015
				Z1	0.101*** ± 0.021
				Z2	0.079*** ± 0.024
			0.95	Ref	0.198 ± 0.029
				Ref1	0.263 ± 0.031
				VIUA	0.234 ± 0.029

				SB	0.237 ± 0.031
				Z1	0.378***▲ ± 0.034
				Z2	0.370***▲ ± 0.043
			1.05	Ref	0.297 ± 0.033
				Ref1	0.259 ± 0.031
				VIUA	0.287 ± 0.031
				SB	0.316 ± 0.034
				Z1	0.167**▲ ± 0.026
				Z2	0.165*▲ ± 0.033
			1.10	Ref	0.120 ± 0.023
				Ref1	0.068 ± 0.018
				VIUA	0.096 ± 0.020
				SB	0.084 ± 0.020
				Z1	0.029** ± 0.012
				Z2	0.039* ± 0.017
			1.15	Ref	0.042 ± 0.014
				Ref1	0.063 ± 0.017
				VIUA	0.029 ± 0.012
				SB	0.037 ± 0.014
				Z1	0.014 ± 0.008
				Z2	0 + 0.056
			1.20	Ref	0.041▲ ± 0.014
				Ref1	0.005* ± 0.005
				VIUA	0.005* ± 0.005
				SB	0 + 0.056
				Z1	0 + 0.056
				Z2	0.016 ± 0.011
			1.25	Ref	0.021 ± 0.010
				Ref1	0.005 ± 0.005
				VIUA	0 + 0.056
				SB	0 + 0.056
				Z1	0 + 0.056
				Z2	0 + 0.056
	mdh-2	2	1.00	Ref	0.800 ± 0.041
				Ref1	0.903 ± 0.035
				VIUA	0.930* ± 0.021
				SB	0.791▲ ± 0.039
				Z1	0.887 ± 0.024
				Z2	0.806 ± 0.045
			1.10	Ref	0.200 ± 0.041
				Ref1	0.097 ± 0.035
				VIUA	0.070* ± 0.021
				SB	0.209▲ ± 0.039

				Z1	0.113 ± 0.024
				Z2	0.194 ± 0.045
	mdh-3	2	1.00	Ref	0.836 ± 0.034
				Ref1	0.903 ± 0.035
				VIUA	0.930* ± 0.021
				SB	0.791▲ ± 0.039
				Z1	0.894 ± 0.023
				Z2	0.805 ± 0.045
			1.10	Ref	0.164 ± 0.034
				Ref1	0.097 ± 0.035
				VIUA	0.070* ± 0.021
				SB	0.209▲ ± 0.039
				Z1	0.106 ± 0.023
				Z2	0.195 ± 0.045
	mdh-4	1	1.00	Ref	1 – 0.067
				Ref1	1 – 0.063
				VIUA	1 – 0.063
				SB	1- 0.063
				Z1	1 - 0.063
				Z2	1 – 0.063
	mdh-5	2	1.00	Ref	0.978▲ ± 0.011
				Ref1	0.923* ± 0.020
				VIUA	0.957 ± 0.014
				SB	0.905** ± 0.021
				Z1	0.932* ± 0.017
				Z2	1 – 0.063
			1.10	Ref	0.022▲ ± 0.011
				Ref1	0.077* ± 0.020
				VIUA	0.043 ± 0.014
				SB	0.095** ± 0.021
				Z1	0.068* ± 0.017
				Z2	0 + 0.056

t-test revealed a significant difference with:

*- Ref site, p<5%; ** - Ref site, p<1%; *** - Ref site, p<0.1%; ▲ - Ref1 site, p<5%; ▲▲ – Ref1 site, p<1%; ▲▲▲ – Ref1 site, p<0.1%
